# Data showing the effects of temperature and time variances on nano-additives treatment of mild steel during machining

**DOI:** 10.1016/j.dib.2018.05.077

**Published:** 2018-05-18

**Authors:** Sunday A. Afolalu, Abiodun A. Abioye, Mfon O. Udo, Olayide R. Adetunji, Omolayo M. Ikumapayi, Samuel B. Adejuyigbe

**Affiliations:** a Mechanical Engineering Department, Covenant University, Ota, Ogun State, Nigeria; b Mechanical Engineering Department, Federal University of Agriculture Abeokuta, Ogun State, Nigeria; c Mechanical and Mechatronics Engineering Department, Federal University of Oye, Oye- Ekiti, Ekiti State, Nigeria

**Keywords:** Nano-additive, Medium carbon steel, Case-hardening, Machining

## Abstract

The effects of temperature and time variances on nano-additives treatment of mild steel during machining was presented in this study. Mild steel of 150 kg mass containing 0.56% carbon was charged into the furnace at melting and pouring temperature of 1539 and 1545 °C respectively. Also charged into the furnace with the mild steel were 0.05% max phosphorous and a bit of sulphur. Thereafter, the sample was cooled and annealed at a temperature of 900 °C for 9 h and then cooled to 300 °C of hardening, normalizing and tempering respectively. The treated samples were then soaked with pulverized in palm kernel shell and barium carbonate (20%) energizer at respective temperatures (800, 850, 900 and 950 °C) and time variances (60, 90 and 120 min) in a muffle furnace. The developed tool was tested on a lathe machine to evaluate its performance. The surface and core hardness, wear resistance and toughness were carried out using the hardness tester, Rotopol–V and impact tester respectively. This is essential for predicting the useful life of the tool in service.

**Specifications Table**

**Table:** 

Subject area	Mechanical Engineering and Materials Engineering.
More specific subject area	Metallurgical Engineering, Production Engineering and Surface Engineering.
Type of data	Table, text and graph.
How data was acquired	Wear and hardness tests were measured using Rotopol –V and Impact tester respectively. The samples were weighed before and after to get the initial weight and grit was fixed at a point for the sample to revolve at a specific time 600 s/10 min. The maximum energy of the machine used was 300 J of Charpy impact and length of the sample= 55 mm, V-Knut was used at point 22.5 mm. The data was taken at particular interval of machining.
Data format	Raw and analyzed.
Experimental factors	Metallographic preparation was conducted. It involved the grinding and polishing of each sample on emery papers with grit size of 30, 160, 300, 600, 1000 and 1200. Other factors are the melting and pouring temperature, the quenching medium, normalizing and normalizing operations and the time (duration) of each operation.
Experimental features	The casting was carried out at melting and pouring temperature of about 1539 and 1645 °C respectively.
	Heat treatments (annealing, hardening, normalizing and tempering) were conducted on the samples at respective temperatures of 900 and 400 °C for 90 °C per hour then hold for 2 h with natural cooling. The samples were further soaked at respective temperatures and time variances of 800, 850, 900 and 950 °C for 60, 90 and 120 min in a muffle furnance. Test was carried out to check the effects on the samples.
Data source location	Federal University of Agriculture, Abeokuta, Ogun-State, Nigeria.
Data accessibility	Data are available within this article

**Value of the data**

• The data for the hardness and toughness of the developed samples can be used to determine the optimum efficiency of case-hardened cutting tools.
• Wear rate and machining test data could be used to predict the performance of any carburized tool during the machining operation.
• The data on the use of nano-additive concentration can be used to determine the accuracy level of the carburization at each temperature and time.
• Also, the dataset could be used to predict the most significant heat treatment parameters.
• The data obtained could be used in investigating the trend in surface and micro hardness profile of carburized cutting tool.

## 1 Data

The study utilized scrap (steel) for casting using Palm Kernel Shell (PKS) as a nano-additives carbon to develop several cutting tool. Compositional analyses before and after the casting was done with melt correction of carbon increase from 0.560 to 0.65 during melting as shown in Table 1. Data of the micro-hardness values in Table 2 present the core of the carburized samples, while the surface hardness values depict the case of the carburized samples. Tests carried out on the treated cutting tool measured its weight loss, wear volume, wear rate, wear resistance and impact/toughness as shown in Table 3, Table 4.

**Table 1:** Data showing chemical composition of mild steel before and after treatment.

Elements	Composition untreated (%)	Composition treated (%)
C	0.550	0.65
Si	0.852	1.22
Mn	0.526	0.334
P	0.040	0.026
S	0.050	0.036
Cr	0.392	4.34
Ni	0.210	0.16
Mo	0.206	0.89
Al	0.022	0.01
W	0.832	1.67
V	0.222	0.393
Co	0.011	0.012
Fe	96.098	90,029

**Table 2:** Summary of micro hardness and surface hardness test.

Sample (s)	Carburization temperature	Holding time (min)	Micro hardness (HR)	Surface hardness (HR)
A	800	60	48	47
B	800	90	46	60
C	800	120	39	62
D	850	60	35	66
E	850	90	53	66
F	850	120	51	68
G	900	60	41	69
H	900	90	48	70
I	900	120	49	71
J	950	60	45	74
K	950	90	55	76
L	950	120	58	89
Control			55	84

**Table 3:** Summary of weight loss, wear volume and wear resistance results.

		Sample	Weight loss(g) (×10exp-3)	Wear volume (cm^3^) (×10exp-5)		Wear resistance (×10 exp-7)	
1	A		18.0		203.0	4.57	
2	B		20.0		358.0	1.83	
3	C		77.0		998.0	4.74	
4	D		14.0		281.0	2.61	
5	E		20.0		360.0	1.83	
6	F		18.0		332.0	2.03	
7	G		38.0		590.0	9.62	
8	H		24.0		410.0	1.52	
9	I		39.0		603.0	9.37	
10	J		87.0		212.0	4.20	
11	K		47.0		706.0	7.77	
12	L		40.0		616.0	9.13	
13	Control		75.0		968.0	2.05

**Table 4:** Energy absorbed test.

S/N	Sample (S)	Energy absorbed (J)
1	A	23.0
2	B	22.0
3	C	32.0
4	D	71.0
5	E	30.0
6	F	50.0
7	G	75.0
8	H	36.0
9	I	20.0
10	J	16.0
11	K	16.0
12	L	24.0
13	Control	17.0

## 2 Experimental design, materials and methods

**Fig. 1:**
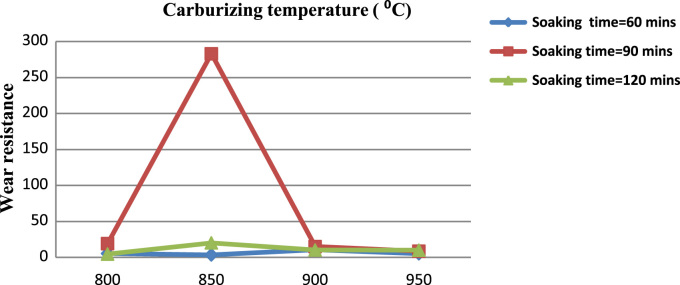
Wear resistance against carburizing temperature.

**Fig. 2:**
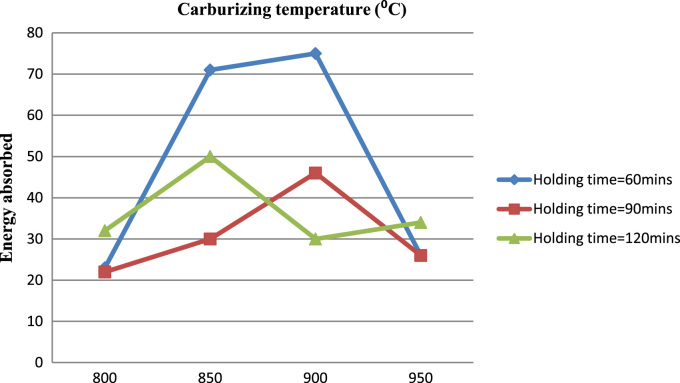
Energy absorbed against carburizing temperature.

The pulverized carbon additive was prepared from palm kernel shell by drying, grinding, milling and sieving. The casting process was carried out with an induction furnace having a maximum temperature capacity of 3000 °C [1], [2], [3], [4], [5], [6]. The furnance was used to melt 150 kg mass of carbon steel at melting temperature of approximately 1539 °C and a pouring temperature of 1545 °C [6], [7], [8]. The samples were annealed at 900 °C at a rate of 90 °C per hour then, it was held for 2 h to cool. Hardening took place at 900 °C a rate of 100 °C /h for 9 h and then it was followed by force-cooling from 900 °C in an oil quenching medium. Thereafter, normalization was conducted at 900 °C at a rate of 100 °C /h for 9 h. Finally, the sample was tempered by heat treatment of 400 °C at a rate of 100 °C/h for 4 h with natural cooling [8], [9], [10], [11], [12]. The treated carbon steel was machined into 24 pieces each of 200 mm×10 mm×10 mm and 20 mm×10 mm×10 mm respectively. Thereafter, they were charged into furnance at temperatures of 800, 850, 900 and 950 °C and duration of 60, 90, 120, 180 min for each stage [12], [13], [14]. In order to evaluate the performance of the developed tool machining tests were carried out on the lathe machine [14], [15], [16]. Furthermore, the hardness tester, Rotopol-V and impact tester were used to conduct surface and core hardness, wear resistance and energy absorbed tests as shown in Fig. 1, Fig. 2 on the developed sample tool [16], [17], [18].
