# Supplementary material for: Data showing the effects of temperature and time variances on nano-additives treatment of mild steel during machining
Source: Data Brief. 2018 May 18;19:456–61. doi: 10.1016/j.dib.2018.05.077 (PMC5997906; doi:10.1016/j.dib.2018.05.077)
Supplement: Supplementary file 1 — Supplementary material [file mmc1.pdf]

Dear Editor,

The work is germane but it was not properly packaged. The problem of recycling waste is a major issue of concern because of the need to save the environment from contamination. Also, the need to conserve resources has become imperative globally because resources are getting scarce. The recycling of waste steel to make useful products for deployment in the field can be a motivating factor for new product development. It will create jobs and indirectly boost the GDP of many a developing nation.

I would suggest acceptance after major revision is done.

All Figures used were not mentioned in the data or experimental design section before they actually came up in the work. The sections were not numbered as per the data in brief template. Grammatical errors were in almost all the sections.

The authors are advised to look at the attached PDF of the article with suggestions and comments on how the work can be improved. The abstract has been rewritten and within the annotated comments in the work. Many corrections were made to improve the work. The authors are advised to read the work properly before submitting all over again to reduce minor errors noticed in the initial work.

Many thanks for the opportunity to serve.

Dr. Olugbenga Omotosho,

Department of Mechanical Engineering,

Covenant University, Ota, Nigeria.

Review response based on DIB questions:

Do the description and data make sense?

Though the data makes sense the description of the data needs improvement.

Do the authors adequately explain its utility to the community?

The utility to the community was properly explained.

Is the protocol for generating data adequate?

The techniques for generating the data is quite adequate and replicated.

Data format (is it standard? potentially re-usable?)

The format of the data is okay to a large extent and of course re-usable.

Does the article follow the Data in Brief template?

The data in brief template was not completely followed by the authors. The text format was not consistent. In some places it was arial, while in others it was times new roman.

Is the data well documented?

Data seems well documented. However, the grammar was generally poor and proper communication was lacking.
